# Zika Virus Infection after Travel to Tahiti, December 2013

**DOI:** 10.3201/eid2008.140302

**Published:** 2014-08

**Authors:** Torgun Wæhre, Anne Maagard, Dennis Tappe, Daniel Cadar, Jonas Schmidt-Chanasit

**Affiliations:** Oslo University Hospital, Oslo, Norway (T. Wæhre, A. Maagard);; WHO Collaborating Centre for Arbovirus and Haemorrhagic Fever Reference and Research, Hamburg, Germany (D. Tappe, D. Cadar, J. Schmidt-Chanasit);; German Centre for Infection Research, Hamburg (J. Schmidt-Chanasit)

**Keywords:** Zika virus, febrile syndrome, travel, French Polynesia, Tahiti, mosquito, viruses

**To the Editor:** Zika virus (ZIKV), a member of the family *Flaviviridae*, is a mosquito-borne virus that is endemic to Africa and Southeast Asia. ZIKV causes illness that is similar to dengue fever, characterized by joint pain, myalgia, headache, and rash (*1*). ZIKV has caused several recent outbreaks, including one in Micronesia in 2007 (*2*) and one in French Polynesia (≈30,000 cases) ongoing since October 2013 (*3*) and spreading to New Caledonia and Easter Island (*4*). We report the clinical and laboratory findings for a patient with ZIKV infection imported from Tahiti, French Polynesia.

The previously healthy 31-year-old woman from Norway was admitted to the Oslo University Hospital, Norway, on December 13, 2013. Six days earlier, she had returned from a 14-day vacation to Tahiti, where she mainly stayed in the capital, Pape'ete, and took a short trip to the island of Mo'orea. One day after her return to Norway, she experienced fever, intense joint pain, and myalgia. Subsequently, a maculopapular rash developed. At the time of admission, her temperature was 37.7°C, and she had enlarged nuchal lymph nodes; injected conjunctivae; and a maculopapular rash on her trunk, extremities, and face (Technical Appendix). Clinical examination findings were otherwise unremarkable. Laboratory tests showed leukopenia of 2.7 × 10^9^ cells/L (reference range 3.5–10 × 10^9^/L), with mild lymphopenia of 1.0 × 10^9^ cells/L (reference range 1.5.–4.0 × 10^9^/L) and neutropenia of 1.4 ×10^9^ cells/L (reference range 1.5–7.3 × 10^9^/L). No thrombocytopenia or elevated liver enzyme levels were detected. C-reactive protein levels (1.4 mg/L) were within reference range.

Because of the patient’s clinical picture and travel history, an acute ZIKV infection was suspected and several diagnostic tests were ordered. In a serum sample taken 5 days after symptom onset, no IgM or IgG against ZIKV, dengue virus (DENV), Japanese encephalitis virus, yellow fever virus, or chikungunya virus was detected by in-house indirect immunofluorescence (*5*,*6*). Only a weak IgG titer of 1:20 (and no IgM) against tick-borne encephalitis virus was found (cutoff <1:20). Test results for DENV nonstructural protein 1 antigen (Platelia; Bio-Rad, Hercules, CA, USA) and generic flavivirus reverse transcription PCR (RT-PCR) (*6*) were negative. Thus, for increased sensitivity, quantitative ZIKV-specific real-time RT-PCR (*6*) with the AgPath-ID One-Step RT-PCR Kit (Life Technologies, Carlsbad, CA, USA) was performed according to the manufacturer’s instructions, and results were positive. ZIKV RNA load was 1.6 × 10^5^ copies/mL; in vitro–transcribed RNA from a reference plasmid was used as a quantification standard.

Attempts to isolate ZIKV in cell culture failed. Therefore, the serum sample was used to obtain the partial ZIKV genome sequence with primers designed from multiple alignments of partial ZIKV genomes retrieved from databases. Primer sequences used for partial genome amplification of ZIKV are available on request (to J. S.-C.). The partial ZIKV genome (strain Tahiti, GenBank accession no. KJ461621) was successfully amplified from the serum sample, and phylogenetic analysis of an ≈200-bp long genomic fragment of the nonstructural protein 3 gene demonstrated that strain Tahiti clusters within the Asian ZIKV lineages and is closely related to a strain from Malaysia (Figure). 

**Figure:**
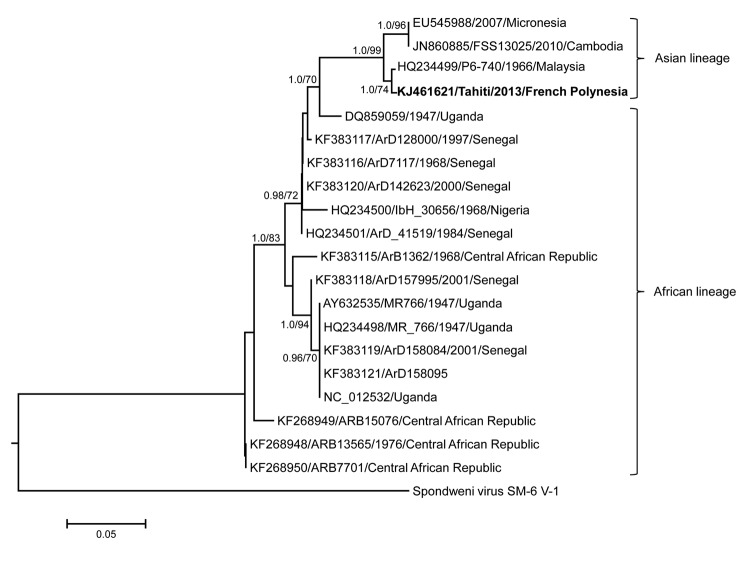
Phylogenetic analysis of partial (≈200 bp) nonstructural protein 3 gene sequences of Zika virus strains performed by using maximum-likelihood and Bayesian methods. A substitution model was based on a general time-reversible model with gamma-distributed rate variation and a proportion of invariant sites. Numbers at the nodes represent posterior probability values (clade credibilities >90%) and percentage bootstrap support values (>70%) based on 1,000 replicates. GenBank accession numbers, strain name, year of isolation, and country of origin for sequences used to construct the tree are indicated on the branches. The tree was rooted with Spondweni virus (GenBank accession no. DQ859064). Strain Tahiti (from patient who had traveled to Tahiti, this study) is indicated in boldface. The scale bar represents genetic distance in nucleotide substitutions per site. The lineage of each virus is indicated to the right of the tree.

In a follow-up serum sample collected 36 days after symptom onset, IgG and IgM seroconversion against ZIKV was demonstrated; IgM titer was 1:1,280 and IgG titer was 1:2,560 (cutoff <1:20). In the same sample, low IgG titers against tick-borne encephalitis virus and DENV (1:40 and 1:80, respectively) were noted (cutoffs <1:20). Real-time RT-PCR for ZIKV in this serum sample was negative.

Travel-related imported ZIKV infections have been reported after travel from Thailand to Germany (*6*) and Canada (*7*), from Indonesia to Australia (*8*), and from Senegal to the United States (*9*). Linked to the current outbreak in French Polynesia, infections in 2 travelers who had returned from Bora Bora to Japan have recently been described (*10*). The clinical findings for the patient reported here (fever, rash, arthralgia, myalgia) were similar to those previously reported for patients with imported cases (*6*,*10*). Available laboratory data are meager, but mild thrombocytopenia has been reported for some patients with Zika fever (*10*), but not for others (*6*,*8*). Outbreaks of dengue fever also occur in French Polynesia (*10*), making dengue fever clinically and epidemiologically the most important differential diagnosis. Elevated liver enzymes, which are found in patients with acute dengue fever, are found in some, but not all, patients with Zika fever (*6*,*8*). 

The measured viral load for the patient reported here (5 days after symptom onset) would not be high enough for efficient transmission of ZIKV to susceptible vectors such as *Aedes aegypti* or *Ae. albopictus* mosquitoes (S. Becker, pers. comm.). This finding is consistent with previously reported findings of ZIKV RNA loads of 930–728,800 copies/mL (*2*). However, *Ae. aegypti* and *Ae. albopictus* mosquitoes are not present in Norway; thus, transmission in Norway seems unlikely. ZIKV infection should be considered as a differential diagnosis for febrile dengue fever–like syndromes in travelers who have returned from Southeast Asia and the Pacific region.

Technical Appendix Photograph of patient with Zika virus infection.
