# Supplementary material for: Zika Virus Infection after Travel to Tahiti, December 2013
Source: Emerg Infect Dis. 2014 Aug;20(8):1412–4. doi: 10.3201/eid2008.140302 (PMC4111184; doi:10.3201/eid2008.140302)
Supplement: Technical Appendix — Photograph of patient with Zika virus infection. [file 14-0302-Techapp-s1.pdf]

Article DOI: http://dx.doi.org/10.3201/eid2008.140302

Zika Virus Infection after Travel to Tahiti, December 2013

Technical Appendix

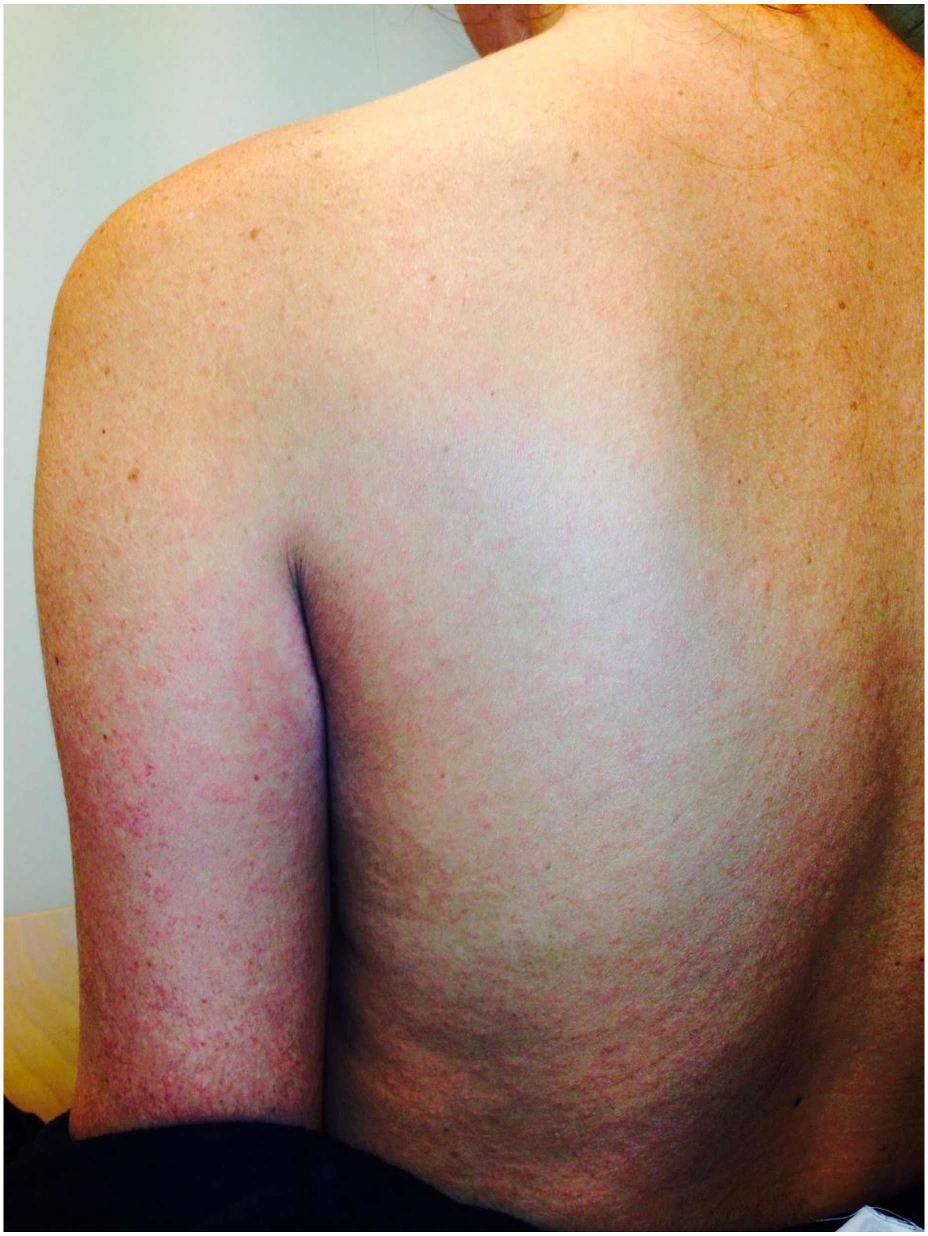

Technical Appendix legend. Maculopapular rash on the trunk and extremities of a patient with Zika virus infection imported from Tahiti, French Polynesia, December 2013.
